# A novel *NFIA* gene nonsense mutation in a Chinese patient with macrocephaly, corpus callosum hypoplasia, developmental delay, and dysmorphic features

**DOI:** 10.1002/mgg3.1492

**Published:** 2020-09-14

**Authors:** Yan Zhang, Cai Mei Lin, Xiao Lan Zheng, Kuerbanjiang Abuduxikuer

**Affiliations:** ^1^ Department of Neurology Xiamen Children's Hospital Fujian China; ^2^ Department of Hepatology Children’s Hospital of Fudan University Shanghai China

## Abstract

**Background:**

*NFIA* gene (OMIM*600727) has been shown to be associated with a syndrome of central nervous system malformations (corpus callosum and ventriculomegaly) with or without urinary tract defects(BRMUTD) (OMIM#613735) with a low incidence.

**Methods and Results:**

** ** We presented the clinical data of a 3‐month‐old Chinese infant with clinical features such as thin corpus callosum, ventriculomegaly, development delay, and dysmorphic features (macrocephaly, hypertelorism, slightly pointed chin, broad forehead, and large ears).** **Genomic DNA was extracted for Trio Whole Exome Sequencing. Preliminary genetic tests revealed one de novo heterozygous nonsense mutation c.220 C>T (p.Arg74Ter) of the *NFIA* gene (NM_005595).

**Conclusion:**

Genetic DNA sequencing is a crucial method for diagnosing BRMUTD. This approach enriches the genotype and spectrum of BRMUTD syndrome and the outcome of the patient.

## Graphical abstract

We presented the clinical data of a 3‐month‐old Chinese infant with clinical features such as thin corpus callosum, ventriculomegaly, development delay, and dysmorphic features. Genomic DNA was extracted for Trio Whole Exome Sequencing. Preliminary genetic tests revealed one de novo heterozygous nonsense mutation of the NFIA gene (c.220C>T/p.Arg74Ter, NM_005595). This approach enriches the genotype and phenotype spectrum of BRMUTD syndrome and the outcome of the patient.

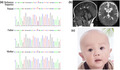

## 1 INTRODUCTION

*NFIA* gene (OMIM*600727) is a transcription factor that belongs to the Nuclear factor I family of dimeric DNA‐binding proteins along with *NFIB*, *NFIC*, and *NFIX*. Nagata, Guggenheimer, Enomoto, Lichy, and Hurwitz (1982) first described Nuclear Factor I (NFI) proteins and their role in vitro, then Wang and Pearson (1985) followed in vivo studies, and Puzianowska‐Kuznicka and Shi (1996) followed studies looking into the *NFIA *gene. NFI proteins play an important role in the development of the central nervous system, including the axon guidance with outgrowth, glial or neuronal cell differentiation, and neuronal migration (Nyboe, Kreiborg, Kirchhoff, & Hove, 2015; Wang & Pearson, 1985). Studies on homozygous Nfia−/− and heterozygous Nfia+/− mice have shown that lack of *NFIA* gene product is associated with the formation of hydrocephalus, the corpus callosum hypoplasia, and urinary tract defects (OMIM#613735) (Bayat, Kirchhoff, Madsen, Roos, & Kreiborg, 2017; Lu et al., 2007). Functional defects on NFI proteins could result in abnormal brain formation, and urinary tract defects (NegishiMiya & Hattori, 2015).

To our knowledge, only six cases of *NFIA* nonsense or frameshift mutation have been reported (Iossifov et al., 2012; Negishi et al., 2015; Revah‐Politi et al., 2017). Iossifov et al. (2012) reported a de novo truncating, heterozygous NFIA mutation (p.Arg83Ter) in a person with autism spectrum disorder, but have not provided information about brain malformation or urinary tract defects. Negishi et al. (2015) described an *NFIA* mutation (p.Pro410HisfsTer32) in a child with interhemispheric cysts, ventricular enlargement, callosal agenesis, and urinary tract defects. Anya Revah‐Politi, Ganapathi, and Bier (2017) described four cases with *NIFA* gene truncating point or frameshift mutations with abnormalities such as ventriculomegaly or hydrocephalus, agenesis of corpus callosum macrocephaly, craniosynostosis, dysmorphic features, Chiari I malformation, seizures, and urinary tract defects. No cases have been reported from East Asia or China, and no disease‐specific treatment is reported. The clinical manifestations of the patients with *NFIA* gene defects described are craniosynostosis, corpus callosum hypoplasia/defect, hydrocephalus/ventricular enlargement, and urinary tract defects.

**FIGURE 1:**
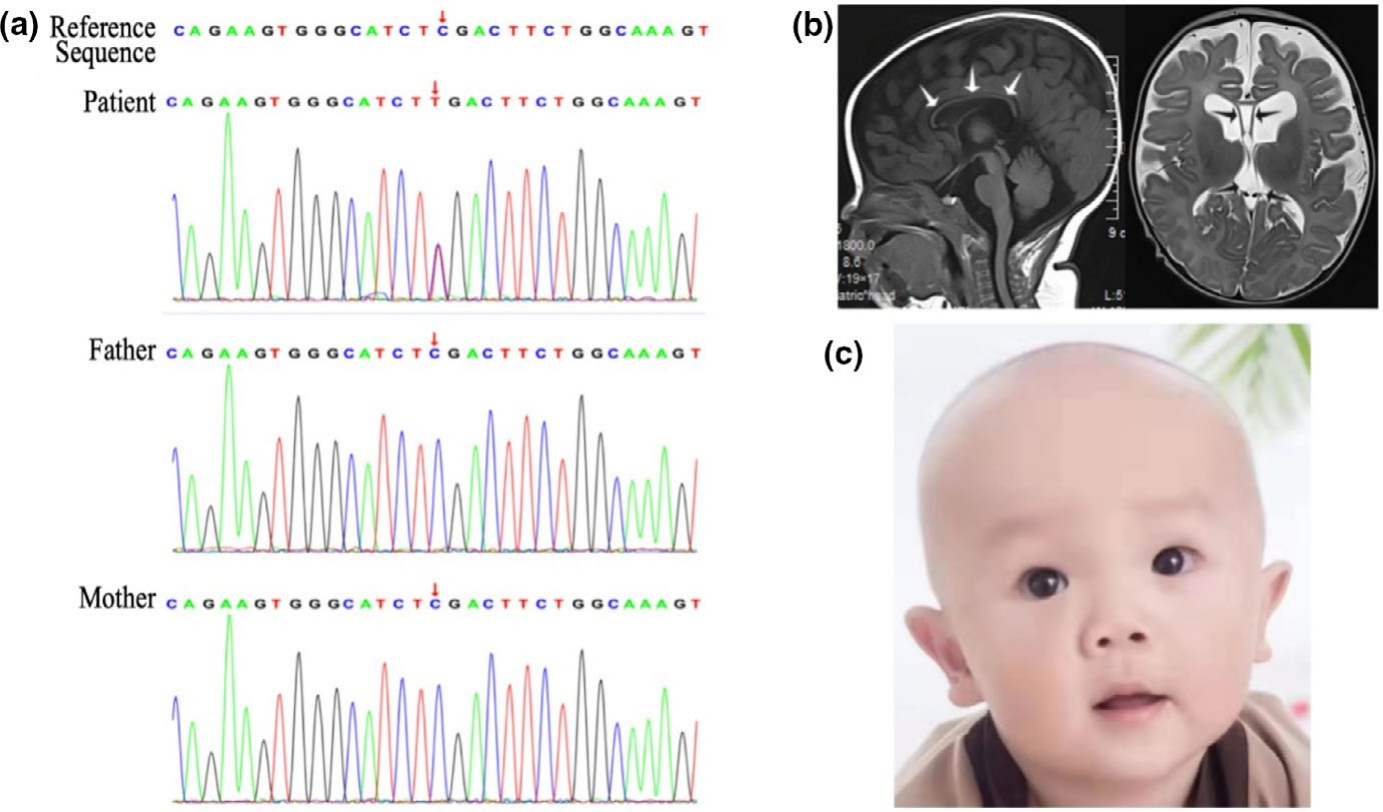
(a) Sanger sequencing confirmation of denovo c.220 C>T(p.Arg74Stop) mutation in the index patient and parents. (b) Left, sagittal T1 weighted image demonstrating a hypoplastic corpus callosum (arrows). Right, T2‐weighted axial image showing ventriculomegaly and a cavum septi pellucidi et Vergae (arrows). (c) Dysmorphic features in the index patient include macrocephaly, hypertelorism, slightly pointed chin, broad forehead, and large ears. (Parental consent was obtained for the publication of personal images)

Here we describe the clinical and molecular features of a Chinese infant with BRMUTD syndrome caused by a novel *NFIA* nonsense mutation, and present clinical features such as thin corpus callosum, ventriculomegaly, development delay, and dysmorphic features (macrocephaly, hypertelorism, slightly pointed chin, broad forehead, and large ears) (Figure 1). He was treated with rehabilitation therapy. His development was improved over time. This study was approved by the ethics committee of the Xiamen Children's Hospital, and written consent for its publication was provided by the patient's parents.

## 2 CASE REPORT

We report a 3‐month‐old male who was the second child of non‐consanguineous parents with unremarkable family history. The ventricular enlargement was suspected from the 28th week of gestation by fetal ultrasonography. He was born after 40 weeks of gestation by cesarean section due to enlargement of the head circumference and post‐term pregnancy. Birth weight was 3700 g, his head circumference was 39.0 cm (>97th centile), and his birth length was 50.5 cm (63rd centile). His Apgar scores were 10, 10, and 10, at 1, 5, and 10 minutes after birth, respectively. Magnetic resonance imaging (MRI) of the brain on the second day of life revealed enlarged bilateral cerebral ventricles, bilateral frontal and temporal brain atrophy, and thin corpus callosum. At the age of 3 month, he was referred to our hospital because he could not hold up his head. He had macrocephaly (head circumference was 43.0 cm (97th centile), normal body length (63.0 cm, 78th centile), hypertelorism, slightly pointed chin, broad forehead, and large ears. The physical examination of heart, lung, abdomen, and nervous system was normal. Complete blood count and arterial blood gas analyses were normal. Serum IgM for cytomegalovirus was positive, but serum and urine samples were negative cytomegalovirus DNA analyses. The result of whole‐genome CNV (copy number variant) analysis was normal. Bilateral ametropia was detected on eye examination, and left ear auditory brainstem response was impaired on hearing screening. Neither renal/ureter, nor skeletal abnormalities were present on the ultrasound examination. DDST (Denver Developmental Screening Test) showed lower DQ (development quotient) (60, normal range >85), and lower MI (mental development index) (70, normal range >85).

The EDTA‐treated blood specimens were collected with informed consent of the patient and his parents. The peripheral white blood cell genomic DNA was extracted using the Blood Genome Column Medium Extraction Kit (Kangweishiji, China) according to the kit instructions. The extracted DNA samples were subjected to quality controling using Qubit 2.0 fluorimeter and electrophoresis with 0.8% agarose gel for further protocol. Liquid hybridization of the genomic DNA was performed using Roche NimbleGen Seq EZ Exome Enrichment Kit V2.0 and Seq EZ Exome Enrichment Kit V2.0 capture probes (Roche, USA), and the target DNA fragments were enriched to construct exome library covering 19,119 genes with whole exons and partial introns. Each enriched region shared 40 Mb of targeted sequences. High‐throughput sequencing was performed by Illumina NovaSeq 6000 series sequencer (PE150), and not less than 99% of target sequence were sequenced. Quality control: Raw data were cleaned after adapters removing, low‐quality reads filtering, and other quality control protocol. Variants calling: The clean data were aligned to the NCBI human reference genome (hg18) using BWA and variants were called using GATK. Samtools and Pindel were used to call SNPs (Single‐Nucleotide Polymorphisms) and indels, respectively. The clean data were then filtered, according to the quality of the sequencing, for further protocol. Variants annotation and prediction: Nonsynonymous substitutions and SNPs with MAF (Minor Allele Frequency) lower than 5% were filtered using SIFT. The function of mutated genes and their pathogenicity were then analyzed referencing to dbSNP, 1000 Genomes Project, ExAC, ESP, OMIM, Swiss‐var, HGMD, ClinVar, and other disease databases. Preliminary genetic tests revealed one de novo heterozygous nonsense mutation of the Nuclear factor I A (*NFIA*) gene (NM_005595, c.220 C>T/p.Arg74Stop) (Table 1). Both parents were screened negative for this mutation.

**TABLE 1:** Features of the seven individuals with NFIA point mutation.

Patients	Patient 1	Patient 2	Patient 3	Patient 4	Patient 5	Patient 6	Patient 7
Reference	Negishi et al. (2015)	Iossifov et al. (2012)	Revah‐Politi (2017)				Current report
Sex and age (years)	Male, 5 years	Male, 5 years	Female, 17 years	Female, 7 years	Female, 35 years	Male, 6 years	Male, 3 months
Country of origin	Japan	USA	UAS	USA	USA	USA	China
Genetic Change (all heterozygous, de novo)	c.1094delC	c.112C＞T	c.159_160dupCC	c.205c>T	c.205c>T	c.205c>T	c.220C>T
Amino acid changes	p.Pro365HisfsTer32	p.Arg83Ter	p.Gln54ProfsTer49	p.Arg69Tet	p.Arg69Ter	p.Arg69Ter	p.Arg74Ter
Abnormal corpus callosum	+	+	+	+	−	+	+
Ventriculomegaly or hydrocephalus	+	+	+	+	+	+	+
Macrocephaly	+	+	+	+	+	+	+
Developmental delay	+	+	+	+	−	+	+
Dysmorphic features	mild macrocephaly, high forehead, and thin upper lip	ND	small hands and feet	Frontal bossing, high forehead	‐	Proximal insertion of thumbs, hemangioma, hypopigmented macule, frontal bossing, high forehead, low anterior hairline, widow's peak,prominent occiput	macrocephaly, hypertelorism, slightly pointed chin, broad forehead, and large ears
Chiari I malformation	−	−	−	+	+	−	−
Seizures	−	−	+	+	+	−	−
Urinary tract defects	+	+	−	+	−	ND	−

Note:

+, trait present; −, trait absent; ND, information not described (trait was not described as present or absent in patient.

He was treated with monosialotetrahexosylganglioside sodium for injection 20 mg per day, 14 days every month for 6 months, and rehabilitation therapy. His development was improved with time. He was able to hold his head up at 6 months, sit alone at 10 months, and hold things to stand at 1 year old. He can call mom and dad with conscious at 1 year old. DDST at 6 months showed a DQ of 83, and an MI of 89. His head circumference was 43.5 cm (50th centile) at 6 months.

## 3 DISCUSSION

All six patients who have *NFIA* nonsense or frameshift mutation have been reported to have macrocephaly, five patients showed abnormalities of corpus callosum, hydrocephalus or ventriculomegaly, and developmental delay. Three patients had seizures and urinary tract defects, including a right renal cyst, cystectasia, left hydronephrosis, congenital renal cyst, frequent urinary tract infections, and urinary retention. Other reported features were obesity, polycystic ovarian syndrome, bilateral radioulnar synostoses, photophobia, headaches, and autism. We summarized the features of the six individuals with *NFIA *point mutation along with our patient in Table 1.

Similar to previous reports in the literature, the patient in our study presented with hypoplasia of the corpus callosum, ventriculomegaly, and developmental delay. Our patient and five out of six previously reported patients have developmental delay. In our case, development delay is improved over time with monosialotetrahexosylganglioside and rehabilitation therapy. Ganglioside is a Glycosphingolipid, which consists of sphingosine, fatty acids, and sugar chain. It exists in the cell membrane of mammals, rich in the cerebral cortex, is an important part of the nerve cell membrane. The species of ganglioside is various, and monosialotetrahexosylganglioside (GM1) is the most important one. GM1 participates in cell recognition, signal transmission, protection of ischemic and anoxic nerve damage, repairment of nerve injury, and restorement of cognitive dysfunction after injury. GM1 can increase the activities of neurotrophic factors, thereby promoting protective effects on the neural system (Yu et al., 2010). Rehabilitation is accepted as the main method for the treatment of developmental delay. Whether the monosialotetrahexosylganglioside therapy without rehabilitation can improve the development delay needs to be confirmed through future studies with more patients.

In summary, we expanded the genotype spectrum of BRMUTD syndrome by presenting the first Chinese infant carrying a novel *NFIA* nonsense mutation and, provided a potential treatment for the development delay for the patient with *NFIA* nonsense mutation. Future reaches are needed, in order to discover optimal drugs and treatment methods for treating children with *NFIA* gene mutation.

## CONFLICTS OF INTEREST

None declared.

## AUTHOR CONTRIBUTIONS

Conceptualization: Yan Zhang, Kuerbanjiang Abuduxikuer; Data curation: Xiao Lan Zheng; Supervision: Cai Mei Lin; Validation: Kuerbanjiang Abuduxikuer; Writing‐original draft: Yan Zhang; Writing‐review & editing: Yan Zhang, Kuerbanjiang Abuduxikuer.
